# The dual role of autophagy in the regulation of cancer treatment

**DOI:** 10.1007/s00726-023-03364-4

**Published:** 2024-02-04

**Authors:** Louis Boafo Kwantwi

**Affiliations:** Department of Pathology, Beckman Research Institute, City of Hope National Medical Center, Duarte, CA USA

**Keywords:** Autophagy, The dual role, Drug resistance, Therapeutic efficacy

## Abstract

As a catabolic process, autophagy through lysosomes degrades defective and damaged cellular materials to support homeostasis in stressful conditions. Therefore, autophagy dysregulation is associated with the induction of several human pathologies, including cancer. Although the role of autophagy in cancer progression has been extensively studied, many issues need to be addressed. The available evidence suggest that autophagy shows both cytoprotective and cytotoxic mechanisms. This dual role of autophagy in cancer has supplied a renewed interest in the development of novel and effective cancer therapies. Considering this, a deeper understanding of the molecular mechanisms of autophagy in cancer treatment is crucial. This article provides a summary of the recent advances regarding the dual and different mechanisms of autophagy-mediated therapeutic efficacy in cancer.

## Introduction

Autophagy is a catabolic and evolutionary conversed process in which cellular components and damaged organelles are degraded or recycled through lysosomal activity (Mizushima 2007). Hence, this process provides cells the ability to adapt to stressful conditions and prevent cellular damage and cell death. (Patergnani et al. 2015; Verma et al. 2021).

In recent times, substantial advances regarding the therapeutic potential of autophagy in cancer have led to several clinical trials expected to block autophagy in cancer. However, the role of autophagy in cancer remains elusive and varies in different contexts and stages of the disease (Singh et al. 2018). Specifically, while autophagy acts as a cytoprotective mechanism to enhance tumor progression, autophagy can promote the sensitivity of tumors to therapeutic agents (Folkerts et al. 2019; Deng et al. 2020; Yu et al. 2021; Ramakrishnan et al. 2012; Kim et al. 2014). Stem from these, to develop an appropriate therapeutic approach based on autophagy modulation in cancer, a comprehensive understanding of the connections existing between autophagy and cancer together with a clear identification of the molecular mechanisms characterizing this axis is important. This review seeks to provide insights into the recent advances regarding the dual role of autophagy in cancer treatment.

## Autophagy-related genes and secreted factors promote drug resistance via autophagy activation

Autophagy-related genes play key roles in therapeutic resistance and hence approaches to silence these genes have recently emerged. For example, suppressing autophagy through genetic knockdown of autophagy‑related 4A (ATG4A) in MCF7 significantly improved the response of breast cancer cells to tamoxifen (Li and Zan 2022). Zhang et al. have proven in breast cancer that adaptor SH3BGRL can potentiate autophagy-induced doxorubicin resistance by promoting PIK3C3 translation and ATG12 stability. Furthermore, the genetic knockdown of either PIK3C3 or ATG12 attenuated autophagy and re-sensitized breast cancer cells to doxorubicin (Zhang et al. 2022). A mechanistic study has revealed that ecotropic viral integration site 1 (EVI1) upregulates ATG7 expression to impede the sensitivity of leukemia cells to imatinib treatment (Niu et al. 2020). In addition, autophagy induction through ATG5 upregulation promotes resistance of gastric cancer cells to fluorouracil (5-FU) (Pei et al. 2018). In a recent study, Tang et al. found that increased expression of Sestrin2 induces autophagy, inhibits apoptosis of tumor cells, and promotes doxorubicin, cisplatin, and methotrexate resistance in osteosarcoma (Tang et al. 2021). Tumor-derived cytokines are important regulators of autophagy-induced drug resistance. For example, autophagy induced by IL6-mediated activation of JAK2 promoted the resistance of colorectal cancer cells to oxaliplatin (Hu et al. 2021). In other experimental settings, the coordinative effect of exosomes and autophagy in drug resistance has been described. According to Pan et al. exosomal circATG4B activated autophagy to induce oxaliplatin resistance in colorectal cancer (Pan et al. 2022) (see Table 1).

**Table 1:** Clinical Trial studies on autophagy modulators

Drug	Cancer Type	Effect on Autophagy	Mode of Action	Trail	Trail ID
CQ	Breast	Inhibits	Lysosome inhibitor	I, II	NCT01023477
HCQ		Inhibits	Lysosome inhibitor	II	NCT01292408
CQ plus taxane		Inhibits	Lysosome inhibitor	II	NCT01446016
HCQ plus ixabepilone		Inhibits	Lysosome inhibitor	I, II	NCT00765765
PF-04691502		Activates	PI3K inhibitor	II	NCT01430585
Triciribine		Activates		II	NCT01697293
Rapamycin plus trastuzumab		Activates	mTORC1inhibitor	II	NCT00411788
HCQ	Pancreatic	Inhibits	Lysosome inhibitor	II	NCT01273805
HCQ plus nucleoside analog gemcitabine		Inhibits	Lysosome inhibitor	I, II	NCT01128296
NVPBEZ235		Activates	PI3K inhibitor	II	NCT01628913
Metformin plus chemotherapy		activates	AMPK activator	II	NCT01210911
HCQ	Prostate	Inhibits		II	NCT00726596
HCQ plus docetaxel		Inhibits		II	NCT00786682
Vinblastine		Inhibits		II	NCT00003084
Everolimus		activates	mTOR inhibitor	II	NCT00657982
Everolimus plus pasireotide		activates	mTOR inhibitor	II	NCT01313559
Everolimus plus paclitaxel		activates	mTOR inhibitor	I, II	NCT00574769
Metformin		activates	AMPK activator	II	NCT01433913
HCQ plus rapamycin	Myeloma	Inhibits		Early phase I	NCT01396200
HCQ plus cyclophosphamide and bortezomib	Multiple myeloma	Inhibits		II	NCT01438177
HCQ plus bortizomib	Multiple myeloma and plasma cell neoplasm	Inhibits		I	NCT00568880
HCQ plus mitoxantrone and etoposide	Acute myeloid leukemia	Inhibits		I	NCT02631252
HCQ plus imatinibmesylate	Chronic myeloid leukemia	Inhibits		II	NCT01227135
HCQ plus rapamycin, cyclophosphamide and dexamethasone	Multiple myeloma	Inhibits		I	NCT01689987
Rapamycin	Hepatocellular	Activates	mTOR inhibitor	II	NCT01079767
Everolimus		Activates	mTOR inhibitor	III	NCT01035229
Sorafenib		Activates	mTOR inhibitor	III	NCT00492752
HCQ plus FOLFOX and bevacizumab	Colorectal		Lysosome inhibitor	I, II	NCT01206530
HCQ plus vorinostat		Inhibits	Lysosome inhibitor	I, II	NCT02316340
Everolimus		Activates	mTOR inhibitor	I, II	NCT00522665
MK-2206		Activates	AKT inhibitor	II	NCT01186705
Metformin plus 5-FU		Activates	AMPK activator	II	NCT01941953
Temsirolimus	Ovarian cancer	Activates		II	NCT01460979
Everolimus plus bevacizumab		Activates	mTORC1 inhibitor	II	NCT01031381

## Circulating RNAs promote drug resistance through autophagy activation

Circulating RNAs play an essential role in metastasis, proliferation, apoptosis resistance and chemoresistance through autophagy induction (Yuan et al. 2022; Liu et al. 2019). In breast cancer, Hsa_circ_0092276 was found to promote proliferation, protection of tumors from apoptosis and doxorubicin resistance (Wang et al. 2021). It has also been established that cirRNAs can promote chemoresistance by sponging miRNAs to upregulate downstream genes (Chen et al. 2019). In colorectal cancer, activation of autophagy through the circHIPK3/miR-637/STAT3 pathway suppressed the sensitivity of cancer cells to oxaliplatin (Zhang et al. 2019).

## Long-noncoding RNAs promote drug resistance through autophagy activation

Emerging evidence have demonstrated that long-noncoding RNNs (LncRNAs) participate in autophagy-induced chemoresistance. In ovarian cancer, autophagy induction by lncRNA XIST promoted the resistance of cancer cells to carboplatin treatment (Xia et al. 2022). According to Hu et al. induction of autophagy via LINC00641/miR‑582‑5p axis promotes the resistance of gastric cells to oxaliplatin (Hu et al. 2020). In a similar study by Hou et al. LINC00963-induced autophagy promoted oxaliplatin resistance (Hou et al. 2021). Relatedly, activation of autophagy through H19/SAHH/DNMT3B promoted resistance of breast cancer cells to tamoxifen (Wang et al. 2019). Zhou et al. investigated how lncRNA PVT1 regulates autophagy-induced gemcitabine in pancreatic cancer cells and showed that PVT1 activated Wnt/β-catenin and enhanced autophagic activity leading to the resistance of cancer cells to gemcitabine (Zhou et al. 2020).

## The tumor microenvironment promotes drug resistance through autophagy activation

Previous studies have demonstrated that immune cells infiltrating tumors can be influenced by interactions in the tumor microenvironment to support tumor progression (Kwantwi 2023b, 2023c; Sheng et al. 2023; Peng et al. 2021; Cai et al. 2020; Kwantwi et al. 2021a; Li et al. 2021). Notably, autophagy induction can shape immune cells to gain protumor functions leading to therapeutic resistance and tumor progression (Bustos et al. 2020; Ishimwe et al. 2020; Jiang et al. 2019; Janji et al. 2018). Emerging evidence indicates that autophagy can promote the tumor-promoting functions of TAMs which makes them attractive targets in TAMs-targeted therapies (Luo et al. 2020; Shan et al. 2017). In laryngeal cancer, autophagy inhibition using chloroquine (CQ) promoted the repolarization of M2 macrophage towards the M1 phenotype. Furthermore, these M1 TAMs showed higher phagocytotic activity towards Hep-2 laryngeal tumor cells and re-sensitized Hep-2 cells to cisplatin treatment, suggesting a role for autophagy activation in cisplatin resistance (Guo et al. 2019). Targeting autophagy through lysosomal inhibitors has shown to be a promising strategy for overcoming therapeutic resistance (Geisslinger et al. 2020; Vyas et al. 2022). In other experimental settings, the link between autophagy activation and impaired antigen presentation and their implication on therapeutic efficacy has been established. According to Li et al. autophagy activation is associated with the degradation of histocompatibility complex class I (MHC-I) and impaired antigen presentation which culminates in anti-PD-1 and anti-CTLA4 resistance in pancreatic ductal adenocarcinoma (Yamamoto et al. 2020). This provides the rationale that a combination of autophagy inhibitors may synergize with immune checkpoint inhibitors to improve treatment response (Li et al. 2017).

PD-L1 is known to suppress the antitumor immunity of the host, promoting tumor progression (Kwantwi et al. 2021b; Kwantwi 2023a). Specifically, tumor intrinsic PD-L1 can promote autophagy induction (Chen et al. 2022; Brogden et al. 2016; Clark et al. 2016) which negatively regulates treatment response. In bladder cancer, tumor cell-intrinsic PD-L1 activated mTORC1 and autophagy to promote cis‐platinum resistance (Zhang et al. 2021a).

## Autophagy activation by stress promotes therapeutic resistance

Endoplasmic reticulum (ER) stress factors which include hypoxia, chemical factors, nutrient deficiency, and intracellular oxygen reactive species can trigger autophagy (Huang et al. 2018; Mrakovcic and Fröhlich 2019). Bouznad et al. recently investigated how IRE1A and XBP-1 regulate the malignant behavior and autophagy-mediated drug resistance in colorectal cancer and revealed that IRE1A-induced hypoxia upregulates XBP-1 and downregulates miR-34a. This promotes EMT and autophagy thereby inducing 5-FU resistance and tumor metastasis, particularly in p53 deficient tumors (Bouznad et al. 2023). Collectively, targeting p53 may be a novel strategy to overcome autophagy-mediated therapeutic resistance in cancer.

Nutrients including but not limited to glucose, amino acids, vitamins, inorganic salts, and lipids are essential for the growth of all cell types and for the maintenance of a steady state in response to adverse environmental conditions (Fan et al. 2022; Butler et al. 2021). Accordingly, since interfering with specific nutrients can be lethal to cells such as cancer, therapeutic strategies to target nutrient dependency in cancer have emerged. This is supported by a recent study conducted to evaluate the relationship between vitamin D and therapeutic resistance. Li et al. uncovered that activation of vitamin D receptor by calcitriol or its analog EB1089 sensitized MCF-7-derived antiestrogen-resistance LCC9 human breast cancer cells to tamoxifen treatment by blocking IRE1α–JNK-mediated autophagy (Li et al. 2021).

## Autophagy activation by Insulin resistance promotes therapeutic resistance

Several lines of evidence strongly suggest that cancer-associated co-morbidities can contribute to the pathophysiology of cancer (Panigrahi and Ambs 2021; Anwar et al. 2021). According to Li et al. autophagy induced by insulin resistance in HCC cells can regulate ER stress to maintain homeostasis thereby promoting the resistance of HCC cells to cisplatin. Furthermore, inhibition of autophagy impaired IR-mediated cisplatin drug in HCC cells (Li et al. 2018a).

## Autophagy activation by microbiota promotes drug resistance

The microbiota is known to play an important role in tumor development and progression (Cheng et al. 2021). Notably, evidence suggest that gut microbiota can modulate the immune response and affect therapeutic outcomes (Sivan et al. 2015; Viaud et al. 2013). Particularly, fusobacterium nucleatum has been implicated in autophagy-mediated therapeutic resistance. According to Yu and colleagues, downregulation of miR-18a and miR-4802 mediated by fusobacterium nucleatum impede the sensitivity of colorectal cancer to oxaliplatin- and 5-FU through TLR4 and MYD-mediated autophagy (Yu et al. 2017).

## Autophagy activation promotes therapeutic efficacy

Besides autophagy-mediated therapeutic resistance already discussed, a huge body of evidence have also established that autophagy activation can improve therapeutic outcomes. Placenta specific 8 (PLAC8) which was first identified through the genomic-wide gene expression analysis of mid-gestation and placentas of embryos is restricted to the spongiotrophoblast layer of the mouse placenta. According to Chen et al. while PLCA8-induced autophagy inhibition promoted breast cancer resistance to adriamycin, activation of autophagy using rapamycin significantly improved the response of breast cancer to adriamycin treatment (Chen et al. 2021).In gastric cancer, autophagy suppression by Rab5a induces resistance of cancer cells to cisplatin treatment but knockdown of Rab5a activates autophagy and enhances the sensitivity of cancer cells to cisplatin treatment (Xu et al. 2018). According to Li et al. autophagy activation by miR-519a improves the sensitivity of glioblastoma to temozolomide treatment (Li et al. 2018b). Furthermore, ADRB2 inhibition in HCC led to enhanced efficacy of HCC cells to sorafenib (Wu et al. 2016). Relatedly, miR-21 inhibition activated autophagy and enhanced the sensitivity of HCC cells to sorafenib treatment (He et al. 2015). High expression of PARD3 in laryngeal squamous cell carcinoma (LSCC) inhibited autophagy and promoted LSCC sensitivity to chemoresistance (Gao et al. 2020). In breast cancer, autophagy activation through fucoidan/mTOR/p70S6K/TFEB pathway enhanced cancer cells to doxorubicin (Zhang et al. 2021b). Zhou et al. showed that chrysin inhibits carbonyl reductase 1 (CBR1) and triggers autophagy-dependent ferroptosis, enhancing the sensitivity of pancreatic cancer cells to gemcitabine (Zhou et al. 2021). In addition to the above, miR‑199a‑5p targeted p62 and inhibited ATG5-mediated autophagy through PI3K/Akt/mTOR pathway activation. This decreased the sensitivity of lung cancer cells to multiple chemotherapeutic drugs, including PTX, taxotere, topotecan, SN38, oxaliplatin, and vinorelbine. However, autophagy activation re-sensitized lung cancer cells to these chemotherapies (Zeng et al. 2021).

Accumulating evidence have shown that autophagy activation can improve treatment response to immune checkpoint inhibitors. It has been found that autophagy activation by V9302, a small-molecule inhibitor of glutamine metabolism, decreases B7H3 expression to boost antitumor immunity of CD8 + T cells. More importantly, anti-PD-1 together with V9302 treatment provided a synergistic effect in patients insensitive to anti-PD-1 therapy (Li et al. 2022a). Autophagy activation can promote the sensitivity of cancer cells to temozolomide by enhancing the polarization of M2 macrophages to M1 phenotype (Li et al. 2022b). In addition, over-expression of MAGE-A downregulated autophagy which resulted in melanoma resistance to CTLA-4 blockade (Shukla et al. 2018), re-enforcing the concept of autophagy activation as a valuable approach to increase the efficiency of immunotherapies.

## Conclusion

Autophagy has become a hotspot in cancer research given its dual role in therapeutic responses. The evidence provided in his review suggests that inhibiting or activating autophagy for therapeutic purposes needs careful consideration. Either case can positively or negatively impact treatment efficacy. Concerning this difficulty, it remains unclear when and how autophagy must be activated or blocked to increase treatment efficiency in patients. However, although autophagy inhibitors would benefit patients with upregulated autophagy, while autophagy activation would be efficient for patients with downregulated autophagy, this approach requires further investigations and specific evaluation.

## Data Availability

Not applicable.
